# Polypharmacy as a risk factor for hospital admission among elderly using emergency transport

**DOI:** 10.1186/1472-6963-14-S2-P2

**Published:** 2014-07-07

**Authors:** Toshikazu Abe, Nanako Tamiya, Takako Kitahara, Yoshinori Hasegawa, Yasuharu Tokuda

**Affiliations:** 1 Department of Emergency Medicine, Mito Kyodo General Hospital, University of Tsukuba, Mito, Ibaraki, Japan; 2 Department of Health Services Research, Faculty of Medicine, University of Tsukuba, Tsukuba, Ibaraki, Japan; 3 Japan Community Healthcare Organization, Tokyo, Japan

## Background

Aging is an urgent global-scale issue and Japan is the frontrunner of aging. Emergency department (ED) admission of the oldest-old challenges emergency physicians and polypharmacy has been considered as one of its possible risk factors. The aim of this study was to analyze hospital admission among patients aged 85 years and older using ambulance transport regarding its relationship with polypharmacy.

## Materials and methods

A retrospective observational cohort study was conducted on consecutive patients (age >85 years old) with ED transports by ambulance between April to December in 2013, in a community teaching hospital in Japan. Patients with out-of-hospital cardiopulmonary arrest were excluded. Data were collected from computerized records about demographics, chief complaints, vital signs and level of consciousness at arrival, final diagnoses at discharge, and polypharmacy (defined > 5 baseline medications) at outpatient clinics. Primary outcome was requirement for admission to the hospital. We also analyzed symptomatic drug adverse events.

## Results

Of the 3084 adults (≥18 years old) who were transported to our hospital by ambulances, 381 (13%) were aged ≥85 years old; 233 (61%) were women. 261/381 (69%) patients were admitted to the hospital. The mean number of their baseline medication was 6.8±3.9. 250/347 (72%) patients had polypharmacy. 27 (7%) patients had apparent symptomatic drug adverse events. Although apparent drug adverse events were not related to polypharmacy (p=0.392), patients with polypharmacy were more likely to be admitted to the hospital after adjusted for age, gender and vital signs at arrival using multiple logistic regression (odds ratio 2.1 [95% CI, 1.0-4.3], p=0.049). Heart and respiratory rates in vital signs at arrival were also associated with admission (P<0.001 and P<0.001, respectively).

## Conclusions

About 70% of the oldest-old patients using ambulance transport were admitted to the hospital. They had been prescribed a number of baseline medications. These medications caused apparent symptomatic drug adverse events, which was one of the most preventable reasons for admission. Polypharmacy could be one of the major risks for admission at ED in addition to unstable vital signs.

